# Principles to operationalize equity in cancer research and health outcomes: lessons learned from the cancer prevention and control research network

**DOI:** 10.1007/s10552-023-01668-0

**Published:** 2023-02-14

**Authors:** Perla Chebli, Prajakta Adsul, Julie Kranick, Catherine L. Rohweder, Betsy C. Risendal, Emily Bilenduke, Rebecca Williams, Stephanie Wheeler, Simona C. Kwon, Chau Trinh-Shevrin

**Affiliations:** 1 Department of Population Health, Section for Health Equity, NYU Grossman School of Medicine, 180 Madison Avenue, 8th Fl. #8-21A, New York, NY 10016 USA; 2 Department of Internal Medicine, University of New Mexico, Albuquerque, NM USA; 3 Cancer Control and Populations Sciences Research Program, University of New Mexico Comprehensive Cancer Center, Albuquerque, NM USA; 4 Center for Health Promotion and Disease Prevention, The University of North Carolina at Chapel Hill, Chapel Hill, NC USA; 5 Colorado School of Public Health, University of Colorado Cancer Center, Aurora, CO USA; 6 Department of Psychology Denver, University of Colorado Denver, Denver, CO USA

**Keywords:** Health equity, Systemic racism, Population health, Social determinants of health

## Abstract

Reflecting their commitment to advancing health equity, the Cancer Prevention and Control Research Network (CPCRN) established a Health Equity Workgroup to identify and distill guiding principles rooted in health equity, community-engaged participatory research (CBPR), social determinants of health, and racial equity frameworks to guide its collective work. The Health Equity Workgroup utilized a multi-phase, participatory consensus-building approach to: (1) identify recurrent themes in health and racial equity frameworks; (2) capture perspectives on and experiences with health equity research among CPCRN members through an online survey; (3) engage in activities to discuss and refine the guiding principles; and (4) collect case examples of operationalizing equity principles in cancer research. Representatives from all CPCRN centers endorsed nine core principles to guide the Network’s strategic plan: (1) Engage in power-sharing and capacity building with partners; (2) Address community priorities through community engagement and co-creation of research; (3) Explore and address the systems and structural root causes of cancer disparities; (4) Build a system of accountability between research and community partners; (5) Establish transparent relationships with community partners; (6) Prioritize the sustainability of research benefits for community partners; (7) Center racial equity in cancer prevention and control research; (8) Engage in equitable data collection, analysis, interpretation, and dissemination practices; and (9) Integrate knowledge translation, implementation, and dissemination into research plans. Dissemination products, such as toolkits and technical assistance workshops, reflecting these principles will foster knowledge transfer to intentionally integrate health and racial equity principles in cancer prevention and control research.

## Introduction

The year 2020 marked a social, political, and public health reckoning as communities across the United States (US) bore witness to the impact of the COVID-19 pandemic and the disproportionate burden and severity of outcomes among communities already experiencing health and economic disparities. The intersection of the COVID-19 pandemic with the Black Lives Matter Movement also motivated deep personal self-reflection and raised critical public consciousness of the need to address and reduce systemic racism. From social justice and population health perspectives, increasing awareness of, and taking action against systemic racism must occur across all spheres and disciplines of public health and health research. Achieving the vision of population health equity requires a more integrated approach than has previously been attempted in the US—one that leverages authentic community engagement and meaningful partnerships, accounts for the roles of social determinants of health (SDH), and demonstrates a deep commitment to tackling systemic racism at multiple levels of influence within the healthcare system and broader socio-political environment. In this paper, we describe a set of principles that can help achieve these objectives, derived both from the existing literature and from our experience in cancer prevention and control.

Cancer remains a significant public health problem, with 1.8 million Americans diagnosed in 2021 and more than 600,000 expected to die from cancer in 2022 [1, 2]. Cancer outcome disparities in the US are expected to widen among racial and ethnic minoritized and medically underserved groups by 2030 [3, 4]. These disparities persist despite evidence-based guidelines for cancer prevention, screening, and treatment that, when implemented equitably, would otherwise be expected to dramatically reduce group differences in outcomes. In response to this persistent problem, the Centers for Disease Control and Prevention (CDC) and the National Cancer Institute (NCI) established, in 2002, the Cancer Prevention and Control Research Network (CPCRN). The CPCRN is a network of academic, clinical, and community partners whose goals are to: accelerate the uptake of evidence-based strategies in cancer prevention and control in communities; increase implementation and reach in medically underserved populations to reduce disparities; investigate determinants of implementation and programmatic success; and develop the workforce in cancer prevention and control research [5, 6].

The CPCRN convenes a wide array of perspectives and expertise for the common goal of reducing cancer burden [6]. The CPCRN is a ‘thematic research network’ of the CDC’s Prevention Research Centers (PRCs) which are funded to “conduct community-based applied public health research to address chronic diseases and other leading causes of death and disability in the United States” [7]. Building on the strong community partnerships of PRCs, the CPCRN prioritizes community-engaged cancer research to increase the local relevance of the research and facilitate adoption of evidence-based interventions (EBIs). The CPCRN collaborates with local, state, and national partners representing multiple sectors, including community-based organizations, federally qualified health centers, and state and national cancer control coalitions. The CPCRN’s structure fosters cross-center collaboration through multicenter workgroups to advance knowledge around a common theme (e.g., Rural Cancer, Survivorship, Implementation Science) with the goal of supporting the implementation of EBIs. Examples of the wide-ranging issues addressed by CPCRN initiatives were documented by White and colleagues [5] and demonstrate this network’s ability to tackle the complex multilevel etiologies of cancer health disparities.

In many ways, these past several years concretized the historic inequities in SDH. The COVID-19 pandemic highlighted that racial and ethnic minoritized and  medically underserved populations experience a disproportionate burden of morbidity and mortality due to unequal access to and distribution of social resources [8–11]. Simultaneously, the brutal murders of George Floyd, Ahmaud Arbery, Breonna Taylor, and other black men and women in the spring and summer of 2020 resulted in protests across the US, raising public consciousness of systemic racism as a fundamental contributor to poor health outcomes in racially minoritized populations [12, 13].

Decades of research have documented how systemic racism is reflected in highly segregated and densely populated neighborhoods whose residents experience substantial levels of poverty and near-poverty [14–17]. These same neighborhoods are often federally designated as medically underserved areas with shortages of culturally and language-appropriate healthcare providers, as well as lack of access to high quality cancer care compared to more resourced areas. When care is sought, patients sometimes endure long waiting periods for appointments, test results, and unnecessary and expensive testing and care. Furthermore, disparities in health insurance coverage serve as additional barriers to equitable care [18]. A problematic history of racism, poor quality of care, and discrimination in the healthcare system have contributed to normative beliefs and practices that deter care seeking. Feelings of mistrust, disrespect, and loss of control are reinforced by community members’ negative experiences and persistent poor health outcomes. Racial and ethnic minoritized groups often report lower quality of care, lack of respect, less communication rapport, lower trust, and higher perceived discrimination with their providers than non-Hispanic whites. According to the 2020 National Cancer Opinion (NCO) Survey, racial and ethnic minoritized groups, including Black (76%), Hispanic (70%), and Asian Americans (66%), are more likely to believe that racism can impact the healthcare a person receives compared to non-Hispanic whites (53%) [19]. In the Study of Women’s Health Across the Nation (SWAN), Black participants were more likely to report racism as a barrier to breast and cervical cancer screening and unequal access to healthcare (71 vs 47%) compared to non-Hispanic whites [20]. Immigrant status adds an additional barrier to breast, cervical, and colorectal cancer screening for Latinx, Asian/Asian American and other minoritized populations [21, 22]. Financial stress, language barriers, low health literacy, lack of transportation and childcare, and stigma may also limit individuals’ ability to adhere to recommended screening and treatment guidelines [23–26]. Another prominent study demonstrated that even after controlling for socioeconomic status, African American men and women, Asian/Pacific Islander men, and Alaska Native/American Indian men and women still have among the highest rates of cancer mortality [27] compared to their non-Hispanic white counterparts. Persistent inequities in SDH contribute to significant disparities in cancer incidence and mortality between Asian/Pacific Islander and Alaska Native/American Indian populations and other minoritized groups [28, 29]. These inequities span multiple generations and originate from longstanding discriminatory and racist policies and practices, including displacement from homelands and forced acculturation [30].

By mid-summer 2020, the growing momentum to name racism as a root cause of inequities culminated in the CDC declaring racism a serious threat to the public’s health [31]. The National Institutes of Health and National Cancer Institute then issued Requests for Information (RFI) for recommendations to strengthen racial equity, diversity, and inclusion in the biomedical workforce and advance health equity research [32, 33]. Taken together, these initiatives signaled to researchers and research institutions the urgency of translating this equity paradigm into their work in an authentic way. In parallel, the events of the last several years inspired reflective conversations about health equity, systemic racism, and SDH within CPCRN. As a result, we conducted a horizon scan within and across collaborating centers to understand how these concepts were being integrated and operationalized across the network. Then we conducted an environmental scan of the current and extensive literature on equity-related frameworks to identify and distill guiding principles common to CPCRN members to synergize and coordinate efforts around health equity research more meaningfully. The search parameters of the environmental scan spanned a review of literature on CBPR, community engagement, participatory, health equity, racial equity, and racial justice frameworks. In a unified commitment to centering equity in our cancer prevention and control research, the CPCRN established the Health Equity Workgroup in 2020. This group comprised representatives from all eight CPCRN Collaborating Centers, the Coordinating Center, funders, and affiliates (formally defined as faculty members, researchers, or community partners who participate in CPCRN activities, but are not funded as a network center), and was charged with developing actionable guiding principles to work towards health and racial equity in cancer prevention and control research.

Although multiple health and racial equity frameworks exist in the literature, there is less guidance on how to operationalize these frameworks in practice, particularly for implementation science and cancer prevention and control, as they focus on equity-oriented research [34, 35]. This paper describes the participatory and consensus-building approach adopted by the Health Equity Workgroup to: (1) curate health and racial equity frameworks, principles, and operational definitions, (2) vet and finalize these principles with academic and community partners at our annual meetings and via survey/qualitative data collection, and (3) highlight case examples among CPCRN members to illustrate the application of these principles in community and clinical practice.

## Developing guiding principles rooted in equity

The development of principles reflecting SDH, health and racial equity, and participatory approaches included a high-level environmental scan of the academic and gray literature, a cross-center survey of CPCRN members, guided discussions in CPCRN Steering and Workgroup meetings, and consensus-building activities at two Annual CPCRN Meetings. This process was iterative and participatory, with built in feedback loops to ensure the rich expertise, perspectives, and experiences of the CPCRN centers were reflected in the equity principles and their operational definitions.

Initial discussions within the Health Equity Workgroup revolved around frameworks currently used by CPCRN centers to ground their cancer prevention and control research in equity. The most commonly cited frameworks included community-based participatory research (CBPR) [36], SDH [37], and the Minority Health and Health Disparities Research Framework [38] from the National Institute on Minority Health and Health Disparities. Further, CPCRN members noted the need to integrate implementation science frameworks—a core strength of the Network and a reflection of its long history of advancing implementation science in cancer prevention and control. Aligned with the recent calls in implementation science for an explicit focus on health equity, anti-racism, and structural racism, the Health Equity Workgroup sought to further clarify the racial equity paradigm through the additional review of the academic and gray literature [39–41]. The R4P framework [42], the Government Alliance on Race and Equity (GARE) [43], and Greenlining Institute [44] were flagged as important resources to center racial equity across the principles. Members of the Health Equity Workgroup reviewed the aforementioned frameworks and reports and conducted a high-level narrative synthesis to inform the first draft of nine principles common among the Network. These principles were presented to the Health Equity Workgroup and CPCRN Steering Committee to solicit preliminary feedback and guide next steps, with a goal of obtaining network-wide commitment to intentionally integrate these equity principles across CPCRN members and within workgroups.

Next, the Health Equity Workgroup disseminated an online survey to current and former members of the CPCRN, federal agency partners, and affiliate members, to assess whether they currently apply the principle in their center’s work (i.e., “Is this principle applied to your Center’s work?”) and to rate the relevance of each principle for a framework common across the CPCRN (i.e., “Please also rate each principle based on what you perceive as its relevance to an integrated equity framework for the CPCRN” (1 indicating lowest relevance and 5 indicating highest relevance). Respondents were also asked to share case examples from their respective centers, suggest evaluation metrics, propose alternative principles informed by their work or existing frameworks, and provide general feedback. Ratings were analyzed and open-ended responses informed operational definitions of the equity principles. The Health Equity Workgroup presented survey results, the revised set of principles, and their definitions at the CPCRN Annual Meeting in 2021 and engaged in consensus building with attendees to refine and approve each guiding principle. Overall, the 28 survey respondents and 79 Annual Meeting participants endorsed nine health and racial equity principles with minor suggestions for modifications. After the meeting, the recommended changes were incorporated into a revised set of principles.

## Refining the principles through consensus building

**Fig. 1:**
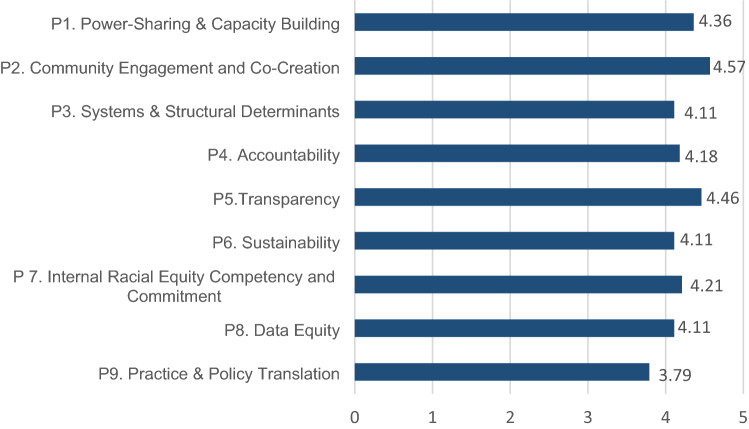
Average relevance of the nine equity principles for the CPCRN

We calculated the average relevance for each principle (score range = 1 [low relevance]–5 [high relevance]) as well as their average application in CPCRN centers. All but one principle (“Practice and Policy Translation” (P9)) were rated 4.11 or above (Fig. 1), reflecting overall high perceived relevance to the CPCRN.

**Fig. 2:**
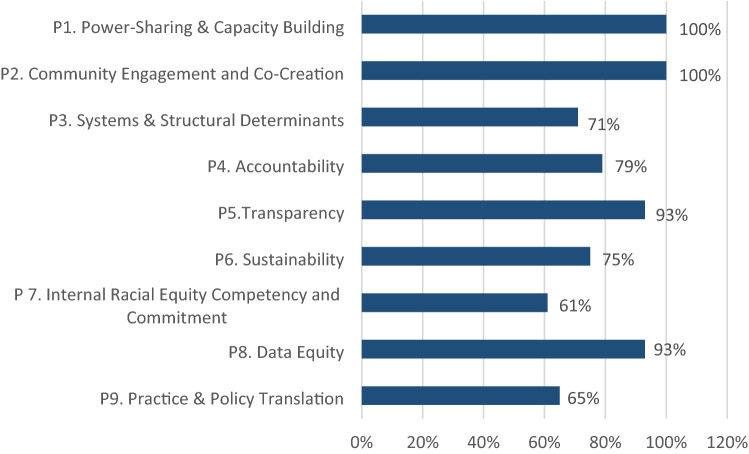
Average application of the nine equity principles

In terms of how each principle was applied in work, at least 60% of respondents felt that the principles were applied in their center’s work (Fig. 2); for some, like power-sharing and capacity building (P1) and community engagement (P2), 100% of respondents stated that these are applied to their work. For others, such as “Competency in and Commitment to Racial Equity” (P7) and “Practice and Policy Translation” (P9), application was lower at 61 and 65%, respectively.

The responses to the open-ended questions provided context for these lower ratings: a few respondents stressed that a racial equity principle was indeed essential, but not yet operationalized in a systematic way within their centers. Furthermore, a few respondents stated that the “Practice and Policy Translation” label was vague and did not stress implementation explicitly. As a result of this feedback, we reconceptualized this principle as “Knowledge Translation, Implementation, and Dissemination” to reflect the growing interest in and commitment to equitable dissemination and implementation, as per the CPCRN strategic plan [45].

**Fig. 3:**
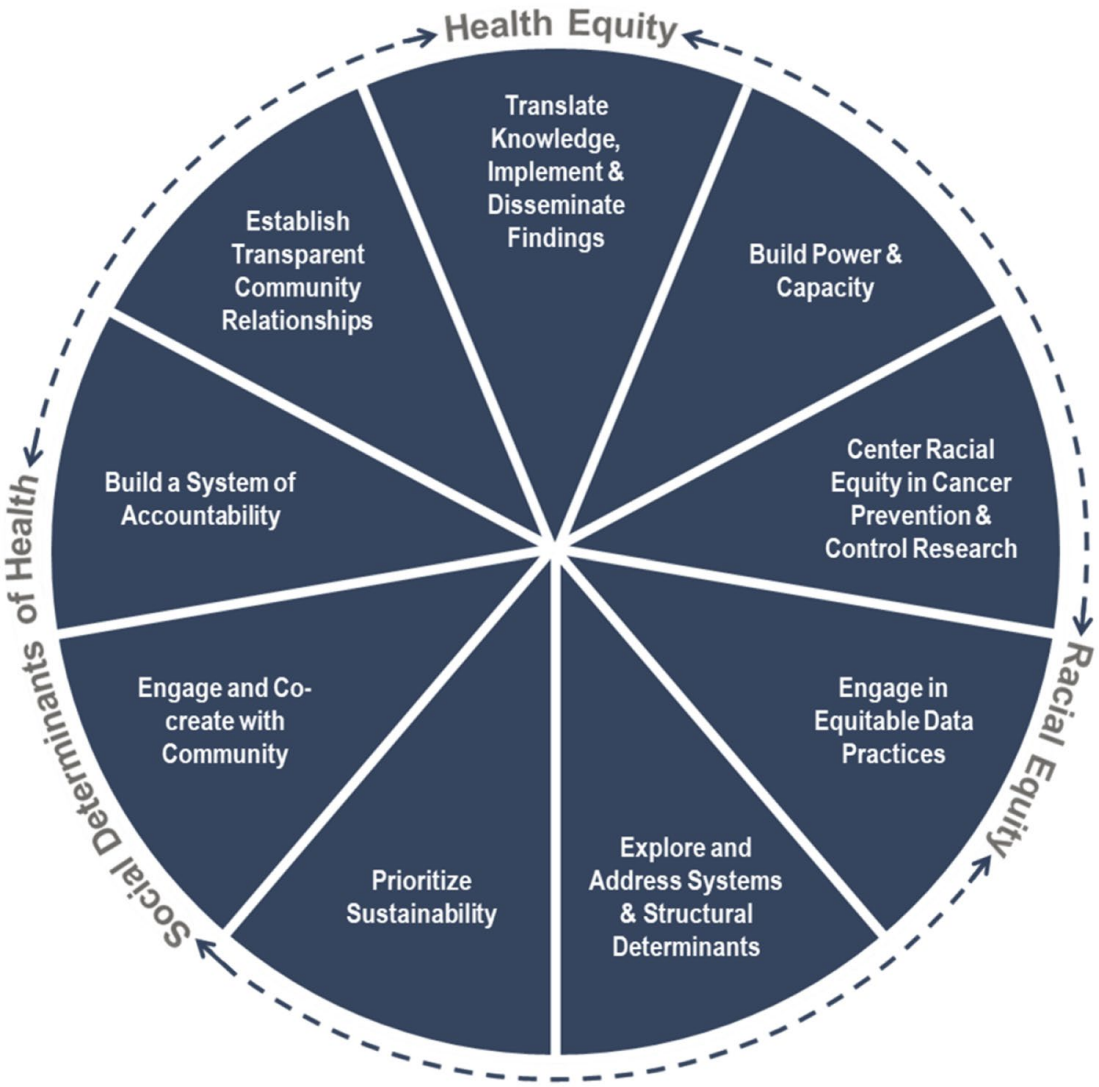
Health and racial equity guiding principles for the CPCRN

Through this process, nine guiding principles were approved, which are anchored in the overarching themes of Health Equity, Racial Equity, and Social Determinants of Health: (1) Engage in power-sharing and capacity building with partners; (2) Address community priorities through community engagement and co-creation of research; (3) Explore and address the systems and structural root causes of cancer disparities; (4) Build a system of accountability between research and community partners; (5) Establish transparent relationships with community partners; (6) Prioritize the sustainability of research benefits for community partners; (7) Center racial equity in cancer prevention and control research; (8) Engage in equitable data collection, analysis, interpretation, and dissemination practices; and (9) Integrate knowledge translation, implementation, and dissemination into research plans (Fig. 3).

## Applying the health and racial equity principles

In addition to specifying the principles, we also focused on their application in cancer prevention and control research to ensure they are both feasible and meaningful. Based on recommendations from the survey and internal discussions within the Health Equity Workgroup, we compiled operational examples of the equity principles as well as case examples from three programs administered by three CPCRN centers (Table 1): (1) the Colorado Cancer Screening Program (CCSP)[46]; (2) Scaling Colorectal Cancer Screening Through Outreach, Referral, and Engagement (SCORE); and (3) New York City Cancer Outreach Network in Neighborhoods for Equity and Community Translation (NYC CONNECT). The CCSP is a statewide program that partners with safety net hospitals and clinics to offer technical assistance and no-cost patient navigation services for cancer screening to the medically underserved of Colorado. CCSP has been funded by the Cancer Cardiovascular and Chronic Pulmonary Disease (CCPD) grants program within the Colorado Department of Public Health and Environment since 2006 to support cancer screening navigation implementation. The SCORE project, funded by the National Cancer Institute’s Accelerating Cancer Screening and Follow Up through Implementation Science (ACCSIS) Program, is designed to address barriers to colorectal cancer (CRC) screening in partnership with Community Health Centers (CHCs). The multilevel intervention includes: (1) centralized mailed fecal immunochemical test (FIT) outreach and (2) patient navigation to follow-up colonoscopy. The study is designed as a type 2 hybrid effectiveness-implementation trial that will assess effectiveness at increasing CRC screening and follow-up rates while also assessing implementation outcomes. Partner CHCs are located in rural counties in western and eastern North Carolina. NYC CONNECT is guided by a community-academic-clinical partnership and aims to improve breast, cervical, and colorectal cancer screening in historically underserved immigrant and minoritized communities in four neighborhoods in New York City. The program uses a trauma-informed approach and leverages community health workers who are embedded in community and clinical settings to deliver, culturally and linguistically tailored cancer education, screening for SDH, and navigation to cancer screening services and social resources (e.g., health insurance, food assistance). In addition, NYC CONNECT works with community partners to explore systemic racism in priority neighborhoods and co-develop and implement locally relevant SDH and racial equity strategies; the goal is to examine whether interventions to account for SDH and reduce the experience of systemic racism may improve cancer screening, wellbeing, and other neighborhood-level social indicators. These three case examples serve to illustrate how the principles were operationalized in existing cancer prevention and control programs serving different populations in various settings.

**Table 1:** CPCRN’s Health Equity guiding principles, definitions, and operationalizations in cancer prevention and control research

Health and racial equity principles	Operational examples	Colorado statewide cancer screening program	SCORE (Scaling Colorectal Cancer Screening Through Outreach, Referral, & Engagement)	NYC CONNECT (Cancer Outreach Network in Neighborhoods for Equity and Community Translation)
P1. Engage in power-sharing and capacity building with partners	• Use transparent, deliberative processes to include and prioritize community and clinical partners voices into cancer prevention and control research • Use consensus building activities with community partners to determine cancer prevention and control priorities for research and identify locally relevant cancer EBIs • Build capacity within the partnerships to engage with academic research and intervention work (i.e., compensate partners for their contribution to the research project; involve community partners in grant writing and manuscript development; support learning exchanges and trainings to cultivate research skills)	• Created a process which allows participating safety net clinic systems to implement cancer screening navigation activities and program components within their site-specific approach to team-based care delivery • Reimbursed partners for patient navigation services • Scheduled regular meetings between clinic partners and project team to review progress and workflow implementation • Connected and supported patient navigators in fundamental navigation training, obtaining continuing education, and placement on Colorado Health Navigation Workforce registry • Collected regular evaluation metrics regarding patient outcome data, reach and impact, and provided feedback to clinic partners to support their ongoing program quality improvement	• Generated the idea for a centralized mailed FIT approach based on feedback from primary care providers; they wanted to screen more patients for CRC but needed support and resources to reach patients who were not up-to-date • Created a centralized mailed FIT model to complement existing visit-based screening and expand reach to patients who don’t come to the clinic regularly • Set priorities with FQHC leadership who were concerned that their communities are located in CRC mortality hotspot • Compensated SCORE FQHCs for their participation in all aspects of the research • Facilitated contributions to publications and presentations from FQHC providers and staff	• Set priorities and developed interventions with community partners, through Neighborhood Action Councils (NACs), including grassroots community organizations serving limited English proficient communities, from the beginning • Used consensus building activities with community partners which identified access to cancer screening, SDH, and food security as top priorities • Participated in joint community and academic training sessions on Trauma-Informed Community Building (TICB) to account for the historical harms and trauma experienced by communities living in disinvested neighborhoods • Meeting regularly and often with community partners (to review emergent findings and guide the project
P2. Address community priorities through community engagement and co-creation of research	• Jointly develop, conduct, and analyze community cancer-related needs and assets assessments in partnership with relevant community partners • Engage community partners in research design, implementation, analysis, and dissemination • Monitor quality of community engagement strategies and partnerships throughout the research project (e.g., through engagement metrics) • Convene and compensate Community Advisory Boards to provide oversight and guidance throughout the research project and identify shared goals and objectives • Integrate community input into recruitment and retention strategies to ensure equitable representation	• Aligned community priorities and preferences for cancer screening and patient navigation with program activities by tailoring program components to local and regional context based on input from partners • Selected and implemented statewide priorities for cancer prevention and control with representatives from the safety net clinic system and their community partners through participation in the development of state cancer plan • Created phone scripts and workflows for wellness and cancer prevention messages and approaches for patient navigators to work from home (or not directly in the clinical setting) during the COVID-19 pandemic • Co-designed elements of program evaluation plan with partners to include metrics of interest to clinic leadership; evaluation metrics revised on regular basis with feedback from partners regarding feasibility of collection and perceived utility of data	• Conducted detailed geospatial analysis showing CRC mortality and location of endoscopy providers to share with communities in northeastern NC • Invested in long-term planning and engagement through multiple consecutive funding mechanisms • Co-developed study protocols with input from providers and staff and tailored them to each site • Adopted a continuous quality improvement style of working with the community health centers. For example, co-created project activities and materials through regular meetings with FQHC staff and providers; solicited recommendations for changes through surveys and interviews • Paused to focus on wellbeing of practice partners when the COVID-19 pandemic hit and started all meetings with check-ins	• Co-created community health needs and resource assessment survey with community partners • Established formal partnership structures at the neighborhood (via NAC) and at the citywide level through the Community Advisory Board to integrate neighborhood-specific priorities and engagement • Conducted a partnership evaluation, grounded in the TICB model, with community partners on an annual basis to continuously improve the quality of engagement, trust building and communication
P3. Explore and address the systems and structural root causes of cancer disparities	• Ground research projects in socioecological models and a life course approach to health, moving beyond individual and interpersonal levels of influence, with a focus on addressing organizational, neighborhood, systems, and/or policy level causes of cancer disparities • Collaborate with community partners to identify root causes of cancer disparities and select potential social, behavioral, systems, and/or policy interventions to address them • Use implementation and intervention mapping to identify strategies to address systems and structural level causes of cancer inequities • Engage multilevel, multisectoral partners to identify and address multilevel determinants of cancer disparities	• Addressed SDH needs by providing health navigation services to reduce barriers to care • Identified and understood multilevel local barriers and facilitators and competing priorities for cancer screening through a program “listening tour” to ensure feasibility and acceptability of program in community • Tailored messaging and outreach approaches to regional perspectives and barriers/facilitators • Focused program recruitment and retention on inclusion of partners who reach and serve under-resourced populations with high barriers related to SDH (safety net, FQHCs, rural and community clinics) • Provided patient navigators with connection to other resources and programs for social needs (2-1-1, housing, food insecurity, resources for non-English speakers)	• Used geospatial mapping of CRC mortality hotspots to guide site selection • Focused on patients not up-to-date with screening who often experience economic distress and/or lack insurance • Negotiated and funded reduced, fixed-fee colonoscopy for uninsured FIT + patients. Built relationships with endoscopy providers to ensure continuity of care • Discussed larger health policy and economic implications such as sustainability of the centralized mailed FIT approach with project partners and statewide coalitions	• Utilized TICB & R4P frameworks which were adapted to reflect systemic racism as the source of collective community trauma • Captured multilevel determinants of cancer outcomes, including an assessment of systemic and interpersonal racism through a community health needs and resource assessment survey • Illuminated factors shaping cancer outcomes and wellbeing, using multi-method formative assessments (i.e., key informant interviews, focus groups, and photovoice) • Tailoring racial equity strategies to each neighborhood to reflect the local context and address relevant determinants of cancer outcomes • Using a community health worker (CHW) navigation model to engage NACs and screen and refer for social needs and other SDH resources
P4. Build a system of accountability between research and community partners	• Formalize network policies and standard operating procedures to explicitly incorporate these principles into collaborative work, and collect equity-related outcome metrics and narrative descriptions via routine progress reporting • Develop/use measures to assess impact of research on cancer disparities and racial equity and report findings to community partners • Develop/use measures to assess impact of research on social, systems, and structural determinants of health and report findings to community partners • Assess, with statistical rigor, both implementation and effectiveness outcomes across sub-populations experiencing disproportionate burden of disease and report sub-population specific findings to community partners and to the research community	• Participated in statewide cancer coalition and patient navigation networks to share data, program experiences, and to facilitate relationships with community and state partners • Provided annual data presentation with funder/state health department officials to share outcomes of program as a state funded program • Annually shared data and outcomes with the Colorado legislature about impact of programming and connection of the funding and work to the medically underserved	• Focused on patients who were unscreened; study sample was stratified on insurance status to reach equal numbers of people with private insurance, Medicaid/Medicare, and no insurance Provided a dashboard with CRC screening metrics, updated regularly, to the practice partners • Presented progress on the study to FQHCs at board meetings and staff/provider meetings; requested feedback on how to make improvements	• Developed norms, values, and commitments between community partners and research team that ground their partnership • Administer annual neighborhood-level surveys to track project impact on cancer screening and SDH and share results with community partners • Hold regular meetings with community partners and the research team • Jointly interpret and share findings from formative assessments with community partners on an ongoing basis
P5. Establish transparent relationships with community partners	• Establish well-delineated roles and expectations and communication norms through memorandums of understanding (MOUs) with community partners • Share project updates and progress in the agreed upon frequency, including outcomes related to cancer disparities and racial equity in plain language briefs	• Tailored Participation Agreements with individual safety net clinic partners and organizations to meet their capacity and needs • Reimbursed partners based on patient navigation services provided with clearly delineated payment structure • Conducted annual meeting where data on program performance was shared with partners, funders, legislators, and community • Presented program evaluation and outcomes at the primary care associations for the safety net clinic partners and connected overall impact on state UDS measures, and Medicaid/Medicare Screening Rates • Co-hosted meetings with Breast and Cervical Programs (WWC/CPED) and existing CHW and PN programs to integrate and ensure authentic relationships and synergy	• Established a business associate agreement with FQHCs so the study team could operate as a third party on behalf of the clinics; this model enabled UNC to deliver FIT kits through a centralized mail process and provide follow-up patient navigation, thus reducing workload for the FQHCs • Clearly outlined roles and expectations through MOUs and other study documents • Co-developed a detailed study charter with community partners	• Jointly created MOUs with community partners tailored to the specific organization • Hold monthly NAC meetings to review data, provide updates, and jointly develop racial equity strategies • Engaged in discussions around expectations, compensation, and resolving disagreements with community partners and the research team
P6. Prioritize the sustainability of research benefits for community partners	• Plan for sustainability of efforts at the outset of project initiation and assess sustainability goals and objectives from community and academic perspectives, including mutual understanding of resources required, opportunity costs of focusing on cancer, and return on investments • Develop cancer-focused interventions in close collaboration with community partners • Align interventions with existing structures/capacity (e.g., practice-based research network, health systems, organizational and systems change) • Build long standing relationships with partners (e.g., continue to serve as technical assistance resource for community partners past grant period)	• Offered sustainability planning TA (sustainability assessment, interpretation of results, strategize goals and implementation) • Gathered case studies and data to support payment transformation models and sustainability (patient-centered medical homes, patient navigating as core service) • Advocated and increased awareness of need for ongoing support for colorectal cancer screening and other preventive screening with state legislature • Built capacity for cancer screening with program webinars and workshops • Maintained long standing partnerships with safety net and rural health primacy care associations, through inclusion in program budgets and planning	• Assessed return-on-investment for FQHCs through economic analyses • Built sustainability and model expansion into the study design and future funding opportunities • Discussed expansion of the centralized mailed FIT model to other FQHCs and primary care practices with statewide partners	• Offered trainings on TICB to community and academic partners • Assessed both needs *and* assets within each neighborhood through formative assessments to build racial equity strategies that leverage existing community assets and resources • Submitting grant applications in collaboration with community partners to sustain NYC CONNECT
P7. Center racial equity in cancer prevention and control research	*Research team-related* • Reflect on individual and research team inherent and unconscious racial biases and implications on research development and design • Support continuous training and education on the role of systemic racism and discrimination on health and cancer care quality and outcomes • Integrate diversity in hiring of research teams to reflect target communities and support recruitment and retention strategies of underrepresented investigators in research teams *Research-related* • Consider the historical and contextual implications of racism and race/ethnicity in cancer data collection and interpretation • Integrate anti-racism approaches to development and implementation of intervention strategies and in the collection of survey and data measures • De-center majority perspectives, shifting a focus and intentionality on examining ‘otherness’ and ‘marginalized social positions’ in the development and implementation of research and in analyzing and interpreting data analyses	• Established programmatic goals driven by existing disparities utilizing data from national sources (BRFSS) and state tumor registry • Sought to integrate diversity in hiring by inclusion of community experience in job descriptions • Recruited and retained community-based organizations with reach and representation in diverse communities as partners to program	• BIPOC research team members were engaged from Eastern Carolina University; they are located in the same region as one of the two community health center systems • Prioritized a diverse research team for SCORE including African American and Latinx faculty and staff	*Research team-related* • Reflected the communities whom we serve within the research team *Research-related* • Ensured diverse community representation in the NACs and CAB • Participate in joint (community partners and research team) TICB trainings • Explored the meanings and manifestations of structural racism and its impact on cancer outcomes and wellbeing through multi-method formative assessments• Translated all study materials and assessments into multiple languages to ensure broad representation of diverse communities • Co-develop and implement racial equity strategies with community partners to address identified priorities for each neighborhood
P8. Engage in equitable data collection, analysis, interpretation, and dissemination practices	• Consult with community partners to determine which cancer outcomes and/or social/structural determinants to measure • Establish co-ownership of data with community partners through data sharing agreements and involve partners in all stages of data collection, analysis, interpretation, and dissemination • Collect and analyze disaggregated data by race/ethnicity • Consider the historical and contextual implications of race/ethnicity in data analysis and interpretation • Choose, collect and analyze measures that reflect adherence to the health and racial equity principles herein	• Collected evaluation metrics that include race, ethnicity, insurance status and other relevant indicators so that equity in reach and impact can be monitored • Co-created evaluation framework to capture information meaningful to participating clinics and communities	• Created data sharing agreements; all study materials are written and delivered in English and Spanish • Accommodated all languages for patients who are FIT positive by using a translation service for patient navigation • Analyzed return rates and positive results for FIT kits by race/ethnicity and other important variables • Focused on insurance status of patient population along with rural location of both community health centers	• Conducted surveys and interviews (i.e., cancer needs and resource assessment and qualitative formative research) in multiple languages (e.g., Spanish, Mandarin, Haitian-Creole) • Sought and incorporated community partners’ feedback on data collection instruments and measures • Collected granular/disaggregated data by race and ethnicity • Engaged diverse informants, reflective of the diverse communities in each neighborhood, in formative qualitative research (i.e., community leaders and members) • Disseminate findings to the NACs and CAB on an ongoing basis
P9. Integrate knowledge translation, implementation, and dissemination into research plans	• Engage and co-create solutions with practice-oriented partners to facilitate translation of evidence-based research and policy into effective community and clinical practice • Integrate dissemination through trusted, community-focused venues in project and engagement goals • Tailor dissemination strategies to partners’ goals, values, literacy, language, and cultural needs • Disseminate and adapt findings to diverse audiences (e.g., policymakers, oncology care providers, primary care providers, community members) • Select implementation strategies that emphasize equitable reach across diverse communities	• Participated in and lead legislative and policy efforts with statewide cancer coalition and policy partners • Trained patient navigators and safety net clinic primary care members in evidence-based approaches for cancer screening • Delivered cultural awareness and culturally sensitive approaches for delivering evidence-based cancer screening interventions through routine trainings and technical assistance in partnership with organizations with diverse representation and expertise • Supported capacity building by regularly disseminating resources and information	• Trained the patient navigator in motivational interviewing skills as the patient perspective is central to the shared decision-making process regarding follow-up care • Leveraged locally developed systems rather than trying to impose a pre-determined, external program design; f example, facilitated the use of local transportation services and built upon a fixed-fee structure already in place for diagnostic colonoscopies • Incorporated continuous feedback on study materials both from providers and patients to accommodate changes in healthcare related to the COVID-19 pandemic	• Trained CHWs to deliver in language, culturally tailored evidence-based approaches for cancer screening • Culturally and linguistically tailored study materials to priority communities • Conduct both implementation and outcome evaluation to determine best practices and lessons learned for CHW interventions and racial equity strategies • Disseminate all findings to a broad and diverse audience including policymakers, providers, academics, community partners, and community members

## Implications for equity in cancer prevention and control

The nine equity principles are informed by seminal and contemporary literature and frameworks on health equity, SDH, racial equity, CBPR, and implementation science [37–42, 47–52]. We provide examples of *how* these guiding principles were applied in CPCRN research with diverse populations in diverse contexts [53, 54]. Operationalizing equity principles through a participatory process in a collaborative network of academic and community partners is an essential step towards achieving population health and reducing cancer disparities. This process paves the path forward and guides the development of evaluation approaches to ensure accountability, quality, and meaningful gains in our collective efforts as a network to achieve cancer equity. Below, we detail next steps and examples of how the equity principles are being integrated into the work of CPCRN centers and workgroups.

First, by acknowledging and measuring inequities and structural racism as fundamental causes of cancer disparities, our work must be followed with deliberate and proactive *actions*. A detailed description of a toolkit outlining steps towards health and racial equity in cancer prevention and control research can be found in Adsul et al. in this supplement [55]. Briefly, the toolkit includes: (1) ways to operationalize each principle; (2) actions taken within the Network to apply the principles; (3) reflection questions to guide future research; and (4) resources and methods for assessment. Aiming to operationalize and illustrate these themes further, the Health Equity Workgroup is currently conducting in-depth interviews with CPCRN centers and their community partners to assemble a comprehensive repository of how all centers are utilizing equity-advancing strategies that could be tailored and implemented across other practice and community settings and population groups.

Second, the CPCRN equity principles will formalize the integration of health equity into the mission and projects of the CPCRN. This commitment to equity is aligned with the priority of the CDC and NCI—CPCRN’s current and previous funding agencies—to address SDH related to cancer disparities. Drawing from the equity principles, the CPCRN proposes several recommendations to diversify the cancer research workforce and improve health equity: (1) increase the diversity of the NIH and biomedical workforce (e.g., Principle 7); (2) involve representatives from minoritized communities on the decision making and scientific review committees (e.g., Principles 1, 2); (3) direct priority funding towards studies that consider the impact of more than one social/structural determinant on health outcomes (e.g., Principle 3); and (4) support long-term community-academic-clinical partnerships for meaningful and impactful health equity research (e.g., Principle 2, 6). In addition, the equity principles are also informing the development of study protocols that prioritize health equity. For example, the Cancer Survivorship Workgroup engaged in a collective reflective process to ensure their planned study on the experiences of racism and patients’ engagement with survivorship care is built around the equity principles from the outset [56].

Third, heeding the warnings against “health equity tourism” [57, 58], we envision the equity principles as foundational for developing *metrics* and *evaluation tools* for the CPCRN to ensure quality research and accountability to our stated commitment to equity. Importantly, members of the CPCRN centers reported that articulating their engagement with each equity principle allowed them to self-reflect, assess their performance, and identify opportunities to further advance equity in their own work. Recognizing that measurement is integral to accountability, the Health Equity Workgroup is developing and validating measures and tools that explicitly assess and evaluate research efforts across CPCRN centers.

## Data Availability

All datasets generated during and/or analyzed during the current study are available from the corresponding author on a reasonable request.

## References

[1] Siegel RL, Miller KD, Fuchs HE, Jemal A (2022). Cancer statistics, 2022. CA Cancer J Clin.

[2] Siegel RL, Miller KD, Fuchs HE, Jemal A (2021). Cancer statistics, 2021. CA Cancer J Clin.

[3] Smith BD, Smith GL, Hurria A, Hortobagyi GN, Buchholz TA (2009). Future of cancer incidence in the United States: burdens upon an aging, changing nation. J Clin Oncol.

[4] Tan DS, Mok TS, Rebbeck TR (2016). Cancer genomics: diversity and disparity across ethnicity and geography. J Clin Oncol.

[5] White A, Sabatino SA, Vinson C, Chambers D, White MC (2019). The Cancer Prevention and Control Research Network (CPCRN): Advancing public health and implementation science. Prev Med.

[6] Ribisl KM, Fernandez ME, Friedman DB, Hannon PA, Leeman J, Moore A (2017). Impact of the cancer prevention and control research network: accelerating the translation of research into practice. Am J Prev Med.

[7] Centers for Disease Control and Prevention (2022) About the PRC Program. Available from: https://www.cdc.gov/prc/about-prc-program/index.htm Accessed on 16 September 2022

[8] Azar KMJ, Shen Z, Romanelli RJ, Lockhart SH, Smits K, Robinson S (2020). Disparities in outcomes among COVID-19 patients in a large health care system in California. Health Aff (Millwood).

[9] Thakur N, Lovinsky-Desir S, Bime C, Wisnivesky JP, Celedon JC (2020). The structural and social determinants of the racial/ethnic disparities in the U.S. COVID-19 pandemic. What's our role?. Am J Respir Crit Care Med.

[10] Lopez L, Hart LH, Katz MH (2021). Racial and ethnic health disparities related to COVID-19. JAMA.

[11] Webb Hooper M, Nápoles AM, Pérez-Stable EJ (2020). COVID-19 and racial/ethnic disparities. JAMA.

[12] Boyd R LE, Weeks L, McLemore M (2020) On racism: a new standard for publishing on racial health inequities Health Affairs Blog [Internet]

[13] Buchman L BQ, Patel J (2020) Black Lives Matter may be the largest movement in U.S. History. New York Times. 7/3/2020

[14] Acevedo-Garcia D, Lochner KA, Osypuk TL, Subramanian SV (2003). Future directions in residential segregation and health research: a multilevel approach. Am J Public Health.

[15] Laveist TA (1993). Segregation, poverty, and empowerment: health consequences for African Americans. Milbank Q.

[16] Riley AR (2018). Neighborhood disadvantage, residential segregation, and beyond-lessons for studying structural racism and health. J Racial Ethn Health Disparities.

[17] Gee GC, Ford CL (2011). Structutral racism and health inequities: old issues. New Directions Du Bois Rev.

[18] Lee DC, Liang H, Shi L (2021). The convergence of racial and income disparities in health insurance coverage in the United States. Int J Equity Health.

[19] National Survey Reveals Racial Differences in Perceptions of Inequities in Health Care and Concerning Delays in Cancer Screenings Amid COVID-19 [press release]. (2020): American Society of Clinical Oncology, 10/1/2020

[20] Jacobs EA, Rathouz PJ, Karavolos K, Everson-Rose SA, Janssen I, Kravitz HM (2014). Perceived discrimination is associated with reduced breast and cervical cancer screening: the Study of Women's Health Across the Nation (SWAN). J Womens Health.

[21] Miller BC, Bowers JM, Payne JB, Moyer A (2019). Barriers to mammography screening among racial and ethnic minority women. Soc Sci Med.

[22] Goel MS, Wee CC, McCarthy EP, Davis RB, Ngo-Metzger Q, Phillips RS (2003). Racial and ethnic disparities in cancer screening: the importance of foreign birth as a barrier to care. J Gen Intern Med.

[23] Adunlin G, Cyrus JW, Asare M, Sabik LM (2019). Barriers and facilitators to breast and cervical cancer screening among immigrants in the United States. J Immigr Minor Health.

[24] Jerome-D'Emilia B (2015). A systematic review of barriers and facilitators to mammography in Hispanic women. J Transcult Nurs.

[25] Honein-AbouHaidar GN, Kastner M, Vuong V, Perrier L, Daly C, Rabeneck L (2016). Systematic review and meta-study synthesis of qualitative studies evaluating facilitators and barriers to participation in colorectal cancer screening. Cancer Epidemiol Biomarkers Prev.

[26] Oh KM, Jacobsen KH (2014). Colorectal cancer screening among Korean Americans: a systematic review. J Community Health.

[27] Ward E, Jemal A, Cokkinides V, Singh GK, Cardinez C, Ghafoor A (2004). Cancer disparities by race/ethnicity and socioeconomic status. CA Cancer J Clin.

[28] Small-Rodriguez D, Akee R (2021). Identifying disparities in health outcomes and mortality for American Indian and Alaska Native populations using tribally disaggregated vital statistics and health survey data. Am J Public Health.

[29] Melkonian SC, Jim MA, Haverkamp D, Wiggins CL, McCollum J, White MC (2019). Disparities in cancer incidence and trends among American Indians and Alaska Natives in the United States, 2010–2015. Cancer Epidemiol Biomarkers Prev.

[30] Warne D, Lajimodiere D (2015). American Indian health disparities: psychosocial influences. Soc Personal Psychol.

[31] Media Statement from CDC Director Rochelle P. Walensky, MD, MPH, on Racism and Health [press release]. (2021), 4/8/21 2021

[32] National Cancer Institute (2021) Request for Information (RFI): Seeking Stakeholder Input on Enhancing Diversity and Inclusion in the Cancer Research Workforce

[33] National Institutes of Health (2021) Request for Information (RFI): Inviting Comments and Suggestions to Advance and Strengthen Racial Equity, Diversity, and Inclusion in the Biomedical Research Workforce and Advance Health Disparities and Health Equity Research

[34] Damschroder LJ (2020). Clarity out of chaos: Use of theory in implementation research. Psychiatry Res.

[35] Kislov R, Pope C, Martin GP, Wilson PM (2019). Harnessing the power of theorising in implementation science. Implement Sci.

[36] Israel BASAJ, Parker EA, Becker AB, Minkler MWN (2008). Critical issues in developing and following community-based participatory research principles. Community-based participatory research for health.

[37] Solar O, Irwin A (2010) A conceptual framework for action on the social determinants of health. Social Determinants of Health Discussion Paper 2. World Health Organization

[38] Alvidrez J, Castille D, Laude-Sharp M, Rosario A, Tabor D (2019). The National Institute on Minority Health and Health Disparities Research Framework. Am J Public Health.

[39] Adsul P, Chambers D, Brandt HM, Fernandez ME, Ramanadhan S, Torres E (2022). Grounding implementation science in health equity for cancer prevention and control. Implement Sci Commun.

[40] Shelton RC, Adsul P, Oh A (2021). Recommendations for addressing structural racism in implementation science: a call to the field. Ethn Dis.

[41] Shelton RC, Adsul P, Oh A, Moise N, Griffith DM (2021). Application of an antiracism lens in the field of implementation science (IS): Recommendations for reframing implementation research with a focus on justice and racial equity. Implement Res Pract.

[42] Hogan V, Rowley DL, White SB, Faustin Y (2018). Dimensionality and R4P: a health equity framework for research planning and evaluation in African American Populations. Matern Child Health J.

[43] Nelson J SL, Ross L, Deng N (2015) Advancing Racial Equity and Transforming Government. A Resource Guide to Put Ideas into Action. The Local and Regional Goverment Alliance on Race and Equity

[44] Creger H (2020). Making racial equity real in research.

[45] Cancer Prevention and Control Research Network (2022) CPCRN Strategic Plan. Available from: https://cpcrn.org/presentations/184/file Accessed on 16 September 2022.

[46] Wolf HJ, Dwyer A, Ahnen DJ, Pray SL, Rein SM, Morwood KD (2015). Colon cancer screening for Colorado's underserved: a community clinic/academic partnership. Am J Prev Med.

[47] Israel BA, Coombe CM, Cheezum RR, Schulz AJ, McGranaghan RJ, Lichtenstein R (2010). Community-based participatory research: a capacity-building approach for policy advocacy aimed at eliminating health disparities. Am J Public Health.

[48] Israel BA, Lachance L, Coombe CM, Lee SD, Jensen M, Wilson-Powers E (2020). Measurement approaches to partnership success: theory and methods for measuring success in long-standing community-based participatory research partnerships. Prog Commun Health Partnersh Res Educ Action.

[49] Israel BA, Schulz AJ, Parker EA, Becker AB (2001). Community-based participatory research: policy recommendations for promoting a partnership approach in health research. Educ Health (Abingdon).

[50] Braveman P (2014). What are health disparities and health equity? We need to be clear. Public Health Reports (Washington, DC : 1974).

[51] Baumann AA, Cabassa LJ (2020). Reframing implementation science to address inequities in healthcare delivery. BMC Health Serv Res.

[52] Chinman M, Woodward EN, Curran GM, Hausmann LRM (2017). Harnessing Implementation Science to Increase the Impact of Health Equity Research. Med Care.

[53] Risendal BC, Hebert JR, Morrato EH, Thomson CA, Escoffery CN, Friedman DB (2021). Addressing COVID-19 using a public health approach: perspectives from the cancer prevention and control research network. Am J Prev Med.

[54] Leeman J, Glanz K, Hannon P, Shannon J (2019). The Cancer Prevention and Control Research Network: Accelerating the implementation of evidence-based cancer prevention and control interventions. Prev Med.

[55] Adsul P, Islam J, Chebli P, Kranick J, Nash S, Arem H, et al. (2023) Identifying research practices towards achieving health equity principles within the Cancer Prevention and Control Research Network. Cancer Causes and Control 10.1007/s10552-023-01674-2 PMC9950692 36826623

[56] Adsul P, Austin JD, Chebli P, Dias EM, Hirschey R, Ravi P, et al. (2023) A clarifying pause: slowing down to do the work of health equity research. Cancer Causes and Control 10.1007/s10552-023-01808-6 PMC10689513 37851185

[57] Lett E, Adekunle D, McMurray P, Asabor EN, Irie W, Simon MA (2022). Health equity tourism: ravaging the justice landscape. J Med Syst.

[58] Nweke N, Isom J, Fashaw-Walters S (2022). Health equity tourism: reckoning with medical mistrust. J Med Syst.

